# Teaching and learning the mental state exam in an integrated medical school. Part I: Student perceptions

**DOI:** 10.1192/pb.bp.113.042655

**Published:** 2014-10

**Authors:** Sarah Huline-Dickens, Eithne Heffernan, Paul Bradley, Lee Coombes

**Affiliations:** 1 Peninsula College of Medicine and Dentistry, Plymouth, UK

## Abstract

**Aims and method** To investigate medical students’ performance at and perceptions of the mental state examination (MSE) at a medical school with a modern integrated curriculum. We undertook an evaluative case study comprising a survey and analysis of performance data. The study is presented in two parts: part 1 discusses the students’ perceptions of the MSE and the teaching, learning and practising of it.

**Results** Most students in the study group considered the MSE an important examination in medicine. Other perceptions grouped in themes are presented. Unsurprisingly, most students found psychiatric attachments the most useful part of the course for learning about the MSE. About a half of students had witnessed an MSE being undertaken in clinical practice.

**Clinical implications** Although students appear to recognise the importance of this examination in medicine, the teaching and learning of it possibly needs greater emphasis in the undergraduate curriculum, and teaching and learning opportunities improved throughout the course.

It is specified in *Tomorrow’s Doctors*^1^ that on qualifying all doctors must be able to perform a mental state examination (MSE) and interpret the findings. The MSE is unique in medicine for combining a humanistic and scientific approach to understanding the patient. However, very little has been written about the use of the MSE, and even less about how it should be taught. Deriving from the ideas of Karl Jaspers, the German philosopher and psychiatrist, who proposed a conceptual framework for this examination in 1912, MSE has been little modified since. Standard textbooks fail to acknowledge the origin of the MSE and there is scarcely any educational research on its use. Despite this, the MSE has attained the status of a defined learning objective within General Medical Council’s *Tomorrow’s Doctors* (unusual in this respect) and has now been written into medico-legal documents (e.g. in the British Medical Association’s interpretation of mental capacity).^2^

Peninsula College of Medicine and Dentistry, also known as Peninsula Medical School, is one of the eight medical schools in the UK founded in the past 12 years. The curriculum at the College is described as integrated with a problem-based curriculum and exposure to clinical medicine from the beginning of the course. The MSE is learned in year 2 of the clinical skills course by means of small-group work with simulated patients. The sessions are facilitated by clinical skills tutors (not psychiatrists) and feedback is given by peers and by the actors. The MSE is then summatively assessed in years 2 and 4 in integrated structured clinical examinations (ISCEs). Every year 2 and year 4 ISCE has a mental health station at which the students must perform an MSE. Anecdotally, studnets had reported that they found the MSE, as assessed by ISCE, more difficult than other ISCE stations. This study was intended to find out whether this was indeed the case, and why.

In this study we asked about students’ perceptions of the MSE and their most helpful experiences in learning about the examination. The study therefore focused on the evaluation of the teaching and learning of the MSE. The ISCE is described in part 2 of this study.

A great deal of literature now exists on students’ attitudes towards psychiatry and there was no intention of replicating this material. We therefore decided to include a single question in the questionnaire to gauge interest in and orientation towards the subject: whether the student would consider a career in psychiatry. This was used as a proxy indicator of attitude towards the specialty.

## Method

### Participants

All year 3 and 5 students in the three clinical sites of the medical school were sampled on the basis that they had undertaken the ISCE at the end of years 2 and 4. Year 5 students will have completed all their psychiatry training by the time they completed the questionnaire: so the data from this group give an overall view of their experience of undergraduate psychiatry. The students in year 3 are at various stages of their training in psychiatry, as the 6 placement weeks all occur in year 3. Students may not have had any experience of psychiatry at all, or may have had all 6 weeks by the time they answer the questionnaire.

The sample was predominantly female (55%) and White (76%), with a modal age of 21 (range 20-40). They comprised 66% of third-year students and almost 28% of fifth-year students. The percentage of female students is commensurate with the national norm (at 56%), but the number of White students at this medical school is slightly higher than the national average of 70%.^3^

### Materials

A review of two validated questionnaires, the Dundee Ready Education Environment Measure^4^ and the Course Experience Questionnaire,^5^ was undertaken to find a suitable means of measuring students’ perceptions of the MSE. However, it was found that these questionnaires were too generic to answer the research questions of this study and it was decided that a new questionnaire was needed that specifically examined attitudes towards the MSE.

A first draft of the questionnaire was devised using the literature from a review and informal discussions with students. Three areas to be measured by the questionnaire were identified: the nature of the MSE, the teaching, learning and practising of the MSE and its assessment by ISCE (this last area is discussed in part 2 of this study).

The style and structure of the questionnaire were carefully considered, following principles lucidly described by Oppenheim.^6^ The cover was designed to inform respondents of the details of the study, to provide them with instructions and to assure them that the information they would contribute would be confidential and anonymous. These measures serve to encourage respondents to complete the questionnaire fully and accurately.^7^

Sections 1-3 included closed-ended items with a fully labelled five-point response scale, which has been shown to lead to high reliability, validity and respondent satisfaction.^8,9^ A ‘don’t know’ option was included in these scales to allow respondents to express non-attitudes or ambivalent attitudes. This is often preferable to forcing them to select a response that they do not truly endorse, as this increases measurement error.^10^

Some items were phrased positively (e.g. ‘I think the MSE is easy to remember’) and some were phrased negatively (e.g. ‘I find the terms used in the MSE difficult to grasp’) to counter respondents who repeatedly tick ‘agree’ without giving due consideration to each question.^11^

Data were also collected through open-ended questions in sections 2-3. This allows respondents to explain their answers to closed-ended questions, and adds depth and richness to the data collected.^10^ As this was an exploratory case study, the following principles were taken into account when considering the likely responses to the open-ended questions:

1. it was likely that only limited categories of information would be given
2. repetition of similar answers was anticipated
3. quotes from the students were likely to be the richest source of information.

These three principles provided the rationale for the management of the data. Preliminary checking of the pilot information confirmed these assumptions, and so a decision was taken to simply thematise the responses manually, in a process of data reduction. This is further described by Miles & Huberman.^12^

The demographic questions were placed at the end of the questionnaire, as these questions are personal and do not appear to relate directly to the purpose of the survey, and may therefore be off-putting for respondents if they appear at the beginning.^11^ The resulting paper and pen questionnaire was administered to participants (the questionnaire is available as an online supplement to this paper).

### Procedure

As an evaluative educational project, the study was granted approval by the chair of the medical school research ethics committee. A pilot study was undertaken with a group of ten students from year 4 (so not included in the final sample of students). The amended questionnaire was administered to consecutive groups of students during scheduled clinical skills sessions. The questionnaires were returned to clinical skills staff. The data were organised using Microsoft Excel 2007 and were analysed using PASW Statistics for Windows, Version 18.0.

## Results

Out of an overall number of 342 students, 229 completed the questionnaire giving a response rate of 67%. Preliminary analysis of the data showed that there were no substantial differences between the responses of the year 3 and year 5 students. Also, there were more than twice as many year 3 than year 5 respondents (152 *v*. 64 respectively), as well as 13 respondents who did not indicate their year of study. Therefore, it was decided that the data should not be divided by year of study but should instead be analysed and reported as a whole.

The results reported in this paper fall into three sections: section 1, perceptions of the nature of the MSE; section 2, the teaching and learning of the MSE in the integrated curriculum of the school; and in addition there are results from a thematic analysis of open questions (derived from section 3 of the questionnaire). The internal consistency reliability of the closed-ended sections of the questionnaire was obtained using Cronbach’s alpha (0.6<α<0.9 is accepted to be adequate):^13^ section 1 (14 items) α = 0.692, section 2 (7 items) α = 0.616, section 3 (5 items) α = 0.619.

### Perceptions of the mental state exam

Responses to questions 2 and 3 (Table 1) indicate an understanding of the importance of the MSE in medicine by the overwhelming majority of students. Most students (69%) did not find the MSE or its terminology to be too complex (question 7). Also, most students (just over 66%) did not find it too intrusive for the patient, but 24% were unsure (question 6).

**Table 1:** Perceptions of the mental state examination, section 1 (*n* = 229)

	%				
Question	Strongly agree	Agree	Don’t know	Disagree	Strongly disagree
1 ‘I would consider a career in psychiatry’	4.4	11.8	23.4	30.6	27.5
					
2 ‘I only need to know about the MSE for the purposes of the ISCE’	0.4	2.2	1.3	50.2	43.7
					
3 ‘The MSE has little connection with anything else in medicine’	0	1.3	3.9	56.8	34.9
					
4 ‘I find the terms used in the MSE difficult to grasp’	1.3	17	17	45.9	16.6
					
5 ‘I think I know when I need to use the MSE and when to use the MMSE’	10	53.3	21.4	9.6	3.9
					
6 ‘I think that the MSE seems too intrusive for the patient’	0	6.6	24	57.2	9.2
					
7 ‘I think the MSE is too complex’	0.9	9.6	18.8	59.0	10.0
					
8 ‘I know which questions to ask in the MSE’	7	47.6	28.4	13.1	1.3
					
9 ‘I feel that I don’t know enough about psychiatric disorders in order to perform the MSE well’	3.1	29.3	26.2	34.9	4.8
					
10 ‘I have seen doctors using the MSE’	10.5	42.8	5.7	24	14.8
					
11 ‘I think the MSE is easy to remember’	3.5	34.9	27.5	27.9	4.4
					
12 ‘I have had sufficient opportunities to practice the MSE on patients in clinical attachments’	3.1	30.1	17.9	33.6	13.1
					
13 ‘I think the MSE is taught well’	1.7	31	34.1	25.8	5.7
					
14 ‘I think there is enough emphasis on/curriculum time devoted to learning about the mental state exam at PCMD’	4.8	34.1	27.5	28.4	3.5

ISCE, integrated structured clinical examination; MSE, mental state examination; MMSE, mini-Mental State Examination; PCMD, Peninsula School of Medicine and Dentistry.

Just over half of the students (54.6%) appear to know which questions to ask, but 14.8% do not (question 8). It can be seen that this question was probably confusing in including a ‘don’t know’ category as it is possible some students selected this option instead. The responses to question 9 - ‘I feel that I don’t know enough about psychiatric disorders in order to perform the MSE well’ - are more equal in distribution. Most students (39.7%) felt that they do know enough about psychiatric disorders to perform well (by disagreeing with the statement), 26.2% were unsure and 32.4% agreed with the statement. In retrospect, however, it is possible that the double negative of the statement confused responses.

Although a third of students indicated that the MSE is easy to remember (just over 38%), almost the same number (32.3%) disagreed with this statement and 27.5% abstained (question 11).

There are two points of interest concerning question 1. The percentage of students agreeing or strongly agreeing with the statement ‘I would consider a career in psychiatry’ is 16.2% (Table 1, question 1). Although this number is in excess of the usually quoted figure of around 4% of students eventually choosing psychiatry as a career,^14^ it is widely recognised that there may be a gap between intentions to pursue psychiatry as a career while at medical school and future career choice.^15^ However, this question was primarily intended as a proxy measure of orientation towards the specialty. Spearman’s rank order correlation coefficient (i.e. Spearman’s rho) revealed that there is a weak but statistically significant positive correlation (*r*_(22)_ = 0.138, *P*<0.05) between responses to question 1 (‘I would consider a career in psychiatry’) and response to question 10 (‘I have seen doctors using the MSE’). This suggests that students who have seen a doctor using the MSE are somewhat more likely to consider a career in psychiatry. Question 1 was not correlated with any of the other questions in section 1.

### Teaching and learning experiences

**Fig 1:**
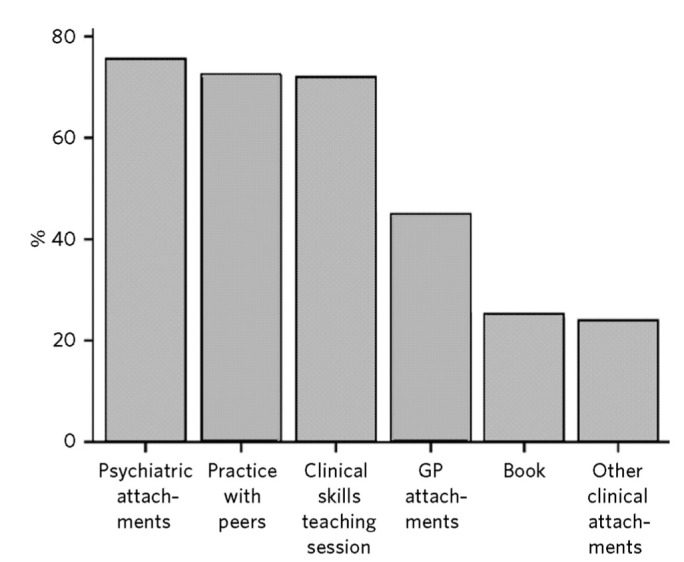
Helpfulness of learning experiences. GP, general practice.

The second section of the questionnaire largely concerned teaching and learning experiences that students had found helpful for learning about the MSE (Fig. 1).

As expected, students found the psychiatric attachments most helpful for learning about the MSE, closely followed by practice with peers and then learning from clinical skills.

A surprising finding was that only half of the students have seen other doctors use the MSE (Table 1), and that most think they have not had sufficient opportunities to practise the MSE on patients in clinical attachments. For example, one student commented: ‘Can we see some non-psychiatric clinicians who actually use it, [accident and emergency] for example or [general practitioners]?’.

Views were mixed about whether the MSE was taught well and about whether there was enough emphasis on learning about the MSE in the curriculum (questions 13 and 14), but with a slight majority thinking there was insufficient emphasis.

The findings from the questions on the most helpful learning experiences were interesting in showing the importance of peer learning to the students (Fig. 1). So although psychiatric attachments were, as expected, cited as the most helpful, this was closely followed by practice with peers and then learning in the clinical skills area. General practice placements, where most students will encounter mental health problems, did not feature highly and neither did the use of books. One possible interpretation of the importance of peer learning is to help in self-management of the difficult experiences cited by some students in the responses to open questions; another is the emphasis on peer learning created by the medical school.

Regarding the responses to the open question 31 about suggestions to help students learn the MSE better (Table 2), 140 students responded to question 31 and the most conspicuous theme emerging was a desire for more practice (31 from 140 responses;).

A total of 50 students (of 229) responded to question 32, comments on MSE teaching at Peninsula Medical School (Table 3), and 21 students to question 33, general comments about the MSE (Table 4). These last two questions are considered together as many of the responses overlapped.

Many of the comments call for a need for more teaching, more practice and tamer actors. It should perhaps be noted that 11 out of 50 responses for question 32 were positive comments. Otherwise new material here includes suggestions by the students on the provision of a student booklet, more teaching placements in year 4, skills for overcoming the students’ anxieties (e.g. ‘Give students confidence - often speaking to psychiatric patients can be scary’), more teaching on interpretation, and a few comments about more consistent judgements on the part of examiners. A wide range of views is given about the quality of teaching, from ‘[MSE] is not really taught’ to ‘I think it is done very well’. In the words of one third-year student: ‘Generally considered the toughest ISCE station with good reason and this should remain the case to encourage greater depth and quality of self-directed learning and practice’.

## Discussion

In undertaking a literature review, few relevant papers were found, as medical student perceptions of the MSE have not been previously studied. Using the terms ‘mental status examination’, ‘mental state exam’ and ‘MSE’ combined variously with ‘medical students’, the databases Cochrane, MEDLINE, PsycINFO, Embase and the educational database ERIC were all searched. In addition, relevant material was inspected from the journals *Medical Education, Clinical Teacher* and *Medical Teacher* from the past 10 years. This procedure is in accordance with accepted protocols: searching for educational studies is acknowledged not to be straightforward by the Best Evidence Medical Education (BEME) group.^16^

Of papers concerning the MSE and the teaching, learning and assessment of psychiatry in medical schools, there are significant limitations: numbers of participating students are often small, terminology differs and outcomes are not clinically relevant or simply have too many variables. Methods of teaching and learning in psychiatry vary between medical schools and are difficult to compare. There appears to be no clear evidence for the most effective way of teaching the MSE. The end-point of being able to apply the MSE to the assessment and diagnosis of real patients in a clinical setting does not appear to have been studied.

A few studies are worthy of mention, however. Karim *et al*^17^ conducted a review of all 35 schools in the UK and Ireland to establish the range of courses in psychiatry available and so this paper has important baseline data. All of the schools provided clinical attachments as a significant part of the teaching programme in psychiatry but methods of teaching were so diverse they were impossible to compare.

The paper by Pohl *et al*^18^ attempts to compare three different methods of teaching the MSE to students (lecture, videotape and simulated patient session). No statistically significant difference was found between students in the three groups when evaluations were performed. However, the effectiveness of the interventions was assessed by multiple choice questionnaires administered before and after each intervention, which would fail to detect any clinically useful outcomes; and there were many variables which were not taken into account, such as teacher influence.

Talley & Littlefield^19^ considered the teaching of the MSE using digital means. This brief description of a change in approach to the teaching of the MSE, substituting online videos of a psychiatrist interviewing an actual psychiatric patient for small-group teaching practice using real faculty members and real patients (which was costly and not sustainable) did actually show an improvement in scores of performance as a result of the new method. There was no change in student satisfaction and the desired reduction in faculty time was achieved. However, the students’ performance was assessed by means of a submitted written-up MSE which arguably fails to test diagnostic reasoning.

Another key paper in the subject literature is that by Goldney & McFarlane,^20^ which is centrally concerned with the problem of defining the skills that need to be assessed in undergraduate psychiatry programmes and investigating the relationship between these skills and academic performance. Essentially, this study tried to compare students’ ratings of psychopathology with their performance using traditional written assessment methods. After analysing the correlations between performance using a number of different assessment methods (MCQ, short answer questions, etc.), the conclusion was there was no correlation between a student’s ability to pass written examinations and their ability to make accurate clinical observations.

**Table 2:** Responses to question 31: ‘Do you have any ideas about how we could help students learn about the MSE more effectively?’ (112 students, 140 responses; maximum 3 suggestions per student)

Response	Frequency, %	Illustrative quotes
Desire for more practice	22.1	More practice with actors doing psychiatric histories, as they are quite different and more intimidating than other histories and exams
		
Desire for more teaching	15.7	More explanation about different word meanings, e.g. thought insertion/ withdrawal
		
Desire for more time	8.6	Allow greater time in year 2 to talk to the simulated patients as we only get one attempt before the ISCE
		
Desire for real patients	6.4	Psychiatric patients instead of actors
		
Desire for involvement of psychiatrists	6.4	MSE practice in clinical skills with psychiatrists there to help and go over language used to describe findings
		
Use of video	5.7	Feedback as groups on videos so can talk about perceptions and compare with each other
		
Need for more clinical teaching	5.7	More clinical practice in hospitals
		
Use of mnemonic	5	I made up the memory aid MAST PLC^a^ which both myself and my friend found extremely useful
		
Use of actors	2.9	Use the actors a lot - very useful
		
Other (not falling strictly into other groupings)	21.4	Simpler scenarios with fewer complications

a. Origin and interpretation unknown.

ISCE, integrated structured clinical examination; MSE, mental state examination.

Although any number of variables and local factors could have biased this finding (e.g. the rating of psychopathology is not absolute, teachers may have had idiosyncratic scoring methods), it is in accordance with other similar work referred to in the paper. The authors conclude that there may be different skills in clinical decision-making: the ability to observe psychopathology, the ability to engage in interpersonal interaction to elicit information, and the ability to apply academic knowledge.

**Table 3:** Responses to question 32: ‘Do you have any comments about how the MSE is taught or examined at PMS?’ (50 students, 50 responses)

Response	Frequency, %	Illustrative quotes
Positive comments	22	Taught well in [clinical skills] and we get to practise with each other
		
Desire for more teaching	16	More teaching on interpretation
		
Comments about actors	10	Simulated patients are often quite unrealistic as it is very difficult to act out someone with a mental illness in a short time period; however, it is still much better than not practising at all
		
Desire for more practice	8	Practise on patients more
		
Need for more resources	6	Would be nice to have a booklet about it as it is badly covered in textbooks
		
Desire for real patients	2	More patient contact would be nice. Don’t really see it utilised unless in psychiatric placement
		
Desire for involvement of psychiatrists	2	Curriculum teaching sessions seemed quite poor compared with teaching from psychiatric consultants
		
Comments about examiners	2	Lots of examiner variations for marking ISCE
		
Comments about clinical teaching	2	Clinical placements on psychiatry are invaluable
		
Other (not falling strictly into other groupings)	30	I think many students underestimate its importance as something you only do with ‘insane’ people Need to emphasise how common mental health problems are Every specialty has to deal with people with mental health issues

ISCE, integrated structured clinical examination; MSE, mental state examination; PMS, Peninsular Medical School.

Owing to the reflective nature of the specialty and difficulties of recruiting into it, there is also a growing literature on the relationship between student attitudes towards psychiatry and whether these might influence their examination performance. The study by Alexander & Eagles^21^ did not find that academic performance was associated with attitudes towards the specialty. However, these attitudes may well influence career choice, and in this respect student reports of the least attractive aspects of the specialty are listed in Box 1.

**Table 4:** Responses to question 33: ‘Do you have any other general comments you would like to make about the MSE?’ (21 students, 21 responses)

Response	Frequency, %	Illustrative quotes
Positive comments	19	I think that the MSE is generally taught well, and is a valuable part of the ISCE
		
Desire for more teaching	9.5	It is a very important skill to learn, there needs to be more effort put into teaching it at clinical skills
		
Comments about examiners	9.5	Some general practitioners [in 4th year pathway assessment] don’t know what it is and think it’s the mini-MSE or the history
		
Desire for more practice	4.7	It needs to be taught carefully and used soon after teaching and often - it is very hard to consolidate MSE, pathways offer little approximation for practice and practising to peers can be confusing if there are gaps in understanding or differences in style
		
Timing	4.7	It is a useful tool but you are expecting us to do a full psychiatric assessment in 20 min and then be capable of presenting this back when we lack experience and knowledge
		
Comments about actors	4.7	You are at the mercy of your actors More training needed for this with respect to how ‘crazy’ to act They sometimes make the station unreasonably difficult
		
Mnemonic	4.7	I found the ABC SMITH from somewhere (not sure where) which I found more useful than ASE PTIC (which is harder to remember)
		
Other (not falling strictly into other groupings)	43	It can be heavy going but has an important place in any psychiatric evaluation

ISCE, integrated structured clinical examination; MSE, mental state examination; ABC SMITH, appearance and behaviour, cognitions, speech, mood, insight, thoughts and hallucinations; ASE PTIC, appearance and behaviour, speech, emotions, perceptions, thoughts, insight, cognitions.

The teaching of psychiatry is thought to have a positive effect on students’ attitudes towards the subject, especially if students are directly involved with patient care, if they see patients respond well to treatment and if they are satisfied with psychiatric staff and patients.^23^ In their study examining the attitudes of students before and after a clinical attachment in psychiatry, Creed & Goldberg^22^ found that students’ (*n*=379) attitudes improved as a result of the attachment and their intentions to pursue psychiatry as a career increased, but there appeared to be no relationship between academic performance (as assessed by both tests of knowledge and clinical exams) and attitudes.

The findings of the present study clearly indicate a reported lack of opportunity to practise the MSE during clinical attachments and witness a doctor using the MSE (only about half of students had). In addition, there were some indications about insufficient exposure to psychiatry and insufficient emphasis on mental health in the curriculum.

There is an association between students seeing a doctor using the MSE and considering a career in psychiatry. However, the direction of causality cannot be deduced from this: it could be that students may be inspired by watching an MSE being performed, or that keener students are more likely to seek opportunities to witness a doctor performing an MSE.

These findings indicate that several practical steps could be taken to improve the teaching and learning opportunities for students, from the provision of resources and materials to more guided practice. The medical school needs to ensure that all students have a chance to witness clinicians using the MSE and provide additional learning resources and support for this important examination. Students need to practise undertaking the examination in clinical settings, be observed and receive feedback on their performance. The study raises important questions about clinicians’ use of the MSE and why students are not witnessing them performing it.

### Limitations

This was an exploratory study investigating students’ attitudes towards the MSE and ways of teaching and learning it at medical school. The questionnaire can be adapted and used by other institutions. However, the questionnaire had limitations, the main one being the inclusion of ‘don’t know’ response options where they may have led to confusion or ambiguity, particularly in the answers to questions 5, 8 and 9. The wording of the questionnaire would need to be refined, and additional work would be needed to validate it. A further limitation is the generalisability of the results as they are derived from one institution. Nevertheless, many schools now have an integrated curriculum and other findings from this study about the perceptions of the MSE are likely to apply to students in other schools too.

**Box 1** Least attractive aspects of psychiatry reported by students, listed by popularity

- Chronic illness unresponsive to treatment
- Aspects of psychiatric patients and their behaviour
- Disagreements between psychiatrists regarding classification and theoretical issues
- Aspects of teaching or the examination
- Clinical psychiatry affects the students themselves
- Psychiatric treatment (electroconvulsive therapy, etc.)
- Staff’s (negative) attitude towards patients
- Staff’s attitude towards students
- Other

Source: Creed & Goldberg^22^

In spite of these limitations some conclusions can be drawn:

- most students in the study group considered the MSE an important examination in medicine
- most students found psychiatric attachments the most useful part of the course for learning about the MSE, closely followed by practice with peers and teaching in clinical skills
- about a half of the students had witnessed an MSE being undertaken in clinical practice.
